# Plasma Biology 2.0

**DOI:** 10.3390/ijms23073684

**Published:** 2022-03-28

**Authors:** Akikazu Sakudo, Yoshihito Yagyu

**Affiliations:** 1 School of Veterinary Medicine, Okayama University of Science, Imabari 794-8555, Japan; 2 Department of Electrical and Electric Engineering, National Institute of Technology, Sasebo College, Nagasaki 857-1193, Japan; yyagyu@sasebo.ac.jp

Plasma biology is a cutting-edge research field that involves plasma technology [1]. Relevant areas of plasma biology include plasma medicine, plasma food science, plasma agriculture as well as plasma environmental science. Much attention has been paid to the use of plasma technology for the development of advanced and innovative new treatments in medicine [2]. The plasma-induced generation of reactive chemical species, such as reactive oxygen species (ROS) and reactive nitrogen species (RNS), is thought to be important for its anticancer or disinfectant activity [2,3,4,5]. However, the mechanisms by which plasma acts in most biological applications remain unclear.

Based on their specific expertise, the Editors of this Special Issue have collated original articles and reviews related to plasma biology. “Plasma Biology 2.0” is a new Special Issue of the *International Journal of Molecular Sciences*, which is a continuation from the previous issue “Plasma Biology” [1]. “Plasma Biology 2.0” comprises five original articles and two reviews reporting various applications of plasma technology as well as discussing the underlying molecular mechanisms.

The article by Benedikt Eggers et al., entitled “Effect of Cold Atmospheric Plasma (CAP) on osteogenic Differentiation Potential of Human Osteoblasts” [6], is related to a previous study by the same authors [7]. The article reports that a cold atmospheric plasma system, Plasma ONE MEDICAL (Plasma MEDICAL SYSTEMS^®^ GmbH) [8], which uses ambient air and a dielectric-barrier discharge (DBD), induces proliferation and mineralization of human calvarial osteoblasts (HCO). In addition, this plasma treatment increases the expression of osteoblast markers of RANK (receptor activator of nuclear factor κB), which is an essential signaling receptor for osteoclastogenesis, and RANKL (RANK ligand). The authors previously reported the effect of plasma on periodontal ligament cells [8], which are related to soft tissue. This study is important because both soft and hard tissue are essential for bone regeneration, which involves the differentiation of osteoblasts. Insights from the study will contribute to the development of potential plasma-based therapeutic tools for bone regeneration.

The article by Dayun Yan et al. entitled “Anti-Melanoma Capability of Contactless Cold Atmospheric Plasma Treatment” [9] demonstrates that a cold atmospheric helium plasma jet can prevent proliferation of melanoma cells under contactless treatment conditions. Dayun Yan et al. found that the helium gas flow rate was an essential parameter for controlling the electromagnetic effect on melanoma cells. Furthermore, they reported that a glass/polystyrene barrier with a thickness of 7 mm could be penetrated by the electromagnetic effect from the cold atmospheric plasma. Overall, they concluded that the contactless cold atmospheric plasma system for antimelanoma treatment is a promising new approach for future cancer therapy.

The article by Fariba Saadati et al., entitled “Patient-Derived Human Basal and Cutaneous Squamous Cell Carcinoma Tissues Display Apoptosis and Immunomodulation following Gas Plasma Exposure with a Certified Argon Jet” [10], reports that treatment with cold atmospheric argon plasma jet kINPen (neoplas GmbH) can induce apoptosis in vitro. Furthermore, they found that gas plasma treatment can induce apoptosis to primary patient-derived squamous cell carcinoma (SCC) and basal cell carcinoma (BCC) tumor samples ex vivo with an associated immunoregulatory effect, such as secretion of chemokines and cytokines.

The article by Catarina Almeida-Ferreira et al., entitled “Cold Atmospheric Plasma Apoptotic and Oxidative Effects on MCF7 and HCC1806 Human Breast Cancer Cells” [11], is a continuation of the study from a previous report [12]. The study compares the plasma responses of two breast cancer cells MCF7 (hormonal-receptor-positive breast cancer cell line) and HCC1806 (triple-negative breast cancer cell line) using cold atmospheric plasma, and then goes on to examine the molecular characteristics of the cell lines. Viable cell numbers decreased following plasma treatment. Interestingly, plasma treatment of MCF cells mainly resulted in apoptosis, whereas HCC1806 cells showed a combination of apoptosis and necrosis. Furthermore, the nitro-oxidative status of the two cell lines showed different patterns. Therefore, the two breast cell lines display distinct characteristics in terms of apoptosis and oxidative stress.

The article by Pradeep Bhartiya et al., entitled “Periodic exposure of plasma-activated medium alters fibroblast cellular homoeostasis” [13], describes a study using plasma-activated medium (PAM), which is prepared by the treatment of medium with a cold atmospheric air plasma jet. The authors investigated the effect of PAM on antioxidative defense mechanisms as well as the intracellular levels of ROS and RNS, together with apoptosis and cell cycle machinery in GM00637 human fibroblast cells. The results suggest that PAM treatment of the cells enhances intracellular accumulation of ROS and RNS. Furthermore, an increase of apoptosis and cell cycle arrest together with upregulation in the expression of antioxidative enzymes, such as glutathione peroxidase (GPx), catalase (CAT), copper/zinc superoxide dismutase (Cu/Zn-SOD), and manganese superoxide dismutase (Mn-SOD), was observed. Overall, this study provides useful information on fibroblast behavior after treatment with PAM or plasma that will aid future therapeutic approaches using PAM and plasma to treat skin diseases.

A review by Kinga Kutasi et al., entitled “Approaches to Inactivating Aflatoxins—A Review and Challenges” [14], outlines detoxification methods for aflatoxins including nonthermal gas plasma treatment as well as discussing the degradation mechanisms of aflatoxins. In addition, the review highlights the potential application of gas plasma for detoxifying agricultural products. The authors also discuss the need to scale-up the plasma delivery system.

A review by Dušan Braný et al., entitled “Effect of Cold Atmospheric Plasma on Epigenetic Changes, DNA Damage, and Possibilities for Its Use in Synergistic Cancer Therapy” [15], summarizes how cold atmospheric plasma induces epigenetic changes and then regulates the expression of noncoding RNAs as well as further changes to their activities. In addition, there is still no consensus on the direct effect of cold atmospheric plasma on inducing DNA damage. These studies are important because cold atmospheric plasma can potentially be used in synergistic treatments alongside other cancer therapies.

Overall, these recent advances in plasma biology will undoubtedly expand our current understanding of its potential application and mechanism of action. Such studies will lead to innovative strategies for the use of plasma technology in a number of different areas.

The Editors are greatly honored to organize this Special Issue for *International Journal of Molecular Sciences*. This Special Issue archives the research of eminent scientists in the field of plasma biology. The Editors wish to thank all the scientists involved in the making of this Special Issue, including the contributors as well as reviewers. The Editors also wish to thank Kaitlyn Wu and the other Editorial staff of the Multidisciplinary Digital Publishing Institute (MDPI) who have worked with dedication and professionalism throughout the publishing process. We hope that readers will be inspired by the collection of articles in this Special Issue.

## Data Availability

Not applicable.
